# Greek Translation and Cultural Adaptation of the Short Version of the Maastricht Utrecht Adherence in Hypertension Questionnaire

**DOI:** 10.7759/cureus.9711

**Published:** 2020-08-13

**Authors:** Ioanna Mylona, Theodoros Tsinopoulos, Anastasios Serbis, Fernando Fernandez-Llimos, Daniela Minarikova

**Affiliations:** 1 2nd Department of Ophthalmology, Aristotle University of Thessaloniki, Thessaloniki, GRC; 2 Department of Organization and Management in Pharmacy, Comenius University of Bratislava, Bratislava, SVK; 3 2nd Department of Pediatrics, Aristotle University of Thessaloniki, Thessaloniki, GRC; 4 Department of Drug Sciences, University of Porto, Porto, PRT

**Keywords:** hypertension, medication adherence, muah-16

## Abstract

Background: The recently published short version of the Maastricht Utrecht Adherence in Hypertension (MUAH) questionnaire (MUAH-16) suggests that MUAH-16 better represents a patient’s adherence to antihypertensive medication than the original MUAH questionnaire.

Objective: The aim of our study was the cultural adaptation and validation of the short MUAH-16 questionnaire in the Greek population.

Methods: 10 patients were involved in the process of translation and cultural adaptation of MUAH-16, providing feedback on the final version, which was then administered to 100 patients. All patients received at least one antihypertensive drug during the last three months and were followed in the Hypertension-24h ABPM ESH Center of Excellence, Outpatient Clinic for the Treatment of Hypertension in the 3rd Internal Medicine Department of Papageorgiou General Hospital of Thessaloniki.

Results: A factor analysis revealed a similar internal structure with four subscales that closely resembled the subscales in the original version of the questionnaire. Internal reliability indexes are equal or better than those of the original subscale structure.

Conclusions: The Greek translation of the MUAH-16 is a good match for the original version with small, cultural differences. More research is needed in order to validate the proposed revised internal structure with a larger sample.

## Introduction

Non-adherence to treatment for hypertension is a significant health issue, with a recent systematic review and meta-analysis of 24 studies claiming a pooled prevalence of non-adherence as high as 31.2% [1]. It is influenced by a number of factors, some of which can be modified [2]. Non-adherence rates have been shown to be associated with age, sex, and race, with lower adherence reported among younger patients [3, 4], males [3], low educational level [3], poor health literacy [3], complexity of daily hypertensive regimen [5], persons of color [6], poor relationship with health care provider [4], emotional response to illness [7], lack of appropriate reminders by the family caregiver [8], depression [9] and culture [10, 11]. A large study of 1367 outpatients found that knowledge of hypertension, patient satisfaction, and coping skills were significantly associated with medication adherence [2]. These findings point to the need on the one hand for patient education, so as to increase one’s knowledge on his/her treatment regimen, and on the other hand for a more effective patient-physician communication so as to improve mutual understanding of specific needs and difficulties.

Wetzels et al. [12] developed a valid and reliable questionnaire for the assessment of adherence problems that hamper intake of medication in patients who are prescribed antihypertensive drugs: the Maastricht Utrecht Adherence in Hypertension (MUAH) questionnaire provides clinicians with information about the causes of a patient’s poor adherence to antihypertensive drugs. Cabral et al. [13] set out to reduce the number of items in the original MUAH questionnaire while retaining satisfactory validity and reliability. The new version, MUAH-16, has reduced the number of items to 16 versus 25 in the original MUAH after an exploratory factor analysis, measuring adherence-related dimensions and global adherence to antihypertensive medication. This measure can be easily applied in a clinical setting, giving health professionals more extended information about the patient’s reasons for poor adherence and allowing the development of more targeted interventions to improve adherence to antihypertensive medication.

Since patients' beliefs and their relationship to medication adherence vary unpredictably across and within countries [14], it is important to validate any measure of patient beliefs regarding antihypertensive treatment when applying it to different cultural settings. The purpose of this study is to translate, culturally adapt and validate the internal structure and reliability of a MUAH-16 version for Greek patients.

## Materials and methods

Study setting and goals

The study took place in the Hypertension-24h ABPM ESH Center of Excellence, Outpatient Clinic for the Treatment of Hypertension in 3rd Internal Medicine Department of Papageorgiou General Hospital of Thessaloniki. The Center of Excellence offers diagnosis and treatment of hypertension by prescribing medication, suggesting dietary plans and follow-up of patients.

The aim of our study was the translation, cultural adaptation and validation of the short MUAH-16 questionnaire in a Greek patient population. This is a cross-sectional pilot study where 10 patients were involved in the initial process of translation and cultural adaptation, while 100 patients participated in the validation of the questionnaire. 

Translation and cultural adaptation

After obtaining permission from the authors, the MUAH-16 was translated and back-translated to Greek, according to the respective World Health Organization (WHO) guidelines for the translation and cultural adaptation titled “Process of translation and adaptation of instruments” [15].

The following steps were followed: 

Step 1: Forward Translation: The translators need to have the following qualifications: native speakers of the respective target language (Greek); knowledge of both English and the target language (Greek); familiarity with the cultures, both of English-speaking countries as well as of the target country (Greece). In this study, two translators with the above-mentioned qualifications translated the MUAH-16 questionnaire independently from each other into the Greek language. The translators were asked to use natural and acceptable language for the broadest audience and to be simple, clear and concise in their formulations. 

Step 2: Expert Panel (Reconciliation of Items): A meeting was held to reconcile the two independent forward translations, which were compared and evaluated in terms of their conceptual equivalence, understanding and clarity of speech relatively to the MUAH-16 English questionnaire.

Step 3: Backward Translation: The backward translation was designed to assess the conceptual equivalence of the reconciled forward translation and the English MUAH-16 questionnaire. The backward translator had the following qualifications: native English speaker; knowledge of both English and the target language (Greek); familiarity with the cultures, both of English speaking countries as well as of the target country (Greece).

Step 4: Review of the Forward and Backward Translation: The review was designed to assess the entire forward backward process in order to provide a final forward translation. Participants in the review procedure were: two members of the research group with good knowledge of both English and Greek; one of the forward translators. The participants reviewed the translation item-by-item by comparing the back-translated items to the English source items. The aim was to develop a final forward translation document.

Step 5: Pre-test (Cognitive Interviews): 10 out of the total 100 patients of different levels of functionality and education, after completing the translated questionnaire, were asked to analyze what they though each question and their corresponding response meant. In this way, it was ascertained that each item retained the original meaning and that no misunderstanding originated during the translation process.

Step 6: Final Version: Patients were included in this study if they fulfilled the following criteria: they were adult patients who attended the outpatient clinic and received at least one antihypertensive drug during the previous three months. The questionnaires were completed from February to August 2019 by 100 patients. The second author distributed the printed version of the questionnaire and expert physicians guided the interviews. The data collection was anonymous and voluntary.

Statistical analysis

Data were analyzed using IBM SPSS® version 24 [16]. All variables were assessed for normality with the Kolmogorov-Smirnov test statistic and comparisons between groups were carried out with appropriate parametric or non-parametric statistics (Student's t-test or Mann-Whitney Z for continuous variables respectively) [17]. Correlations were assessed with the Spearman rs correlation coefficient since the respective comparisons were between non-parametric variables only.

In order to assess factorial invariance between the Greek and the original validation sample, we conducted a factor analysis using the principal axis factoring method [17] and specified the number of component factors to be four, as was the case with the original MUAH-16. The resulting components were subjected to Varimax rotation and the final number of components was evaluated with inspection of a scree plot. The components that were extracted were then evaluated for their meaning and resemblance with the original four-factor structure.

Reliability for each factor was assessed with Cronbach's alpha statistic [18].

## Results

A sample of 100 patients participated in this study: 52 men with a mean age of 54.56 years (SD = 9.264) and 48 women with a mean age of 56.35 years (SD = 1.407). There was no statistically significant difference between the sexes with regards to age, t-test (98) = 0.945, p = 0.347.

We conducted a factor analysis in order to determine the factor structure of the Greek translation of the MUAH-16 in our sample. The Varimax rotation converged in five iterations. The Kaiser-Meyer-Olkin Measure of sampling was 0.719, indicating sufficient items for each factor (adequate value over 0.7). Bartlett's test of sphericity is statistically significant (< 0.001) indicating that the correlation matrix is significantly different from an identity matrix, in which correlations between variables are all zero.

**Figure 1:**
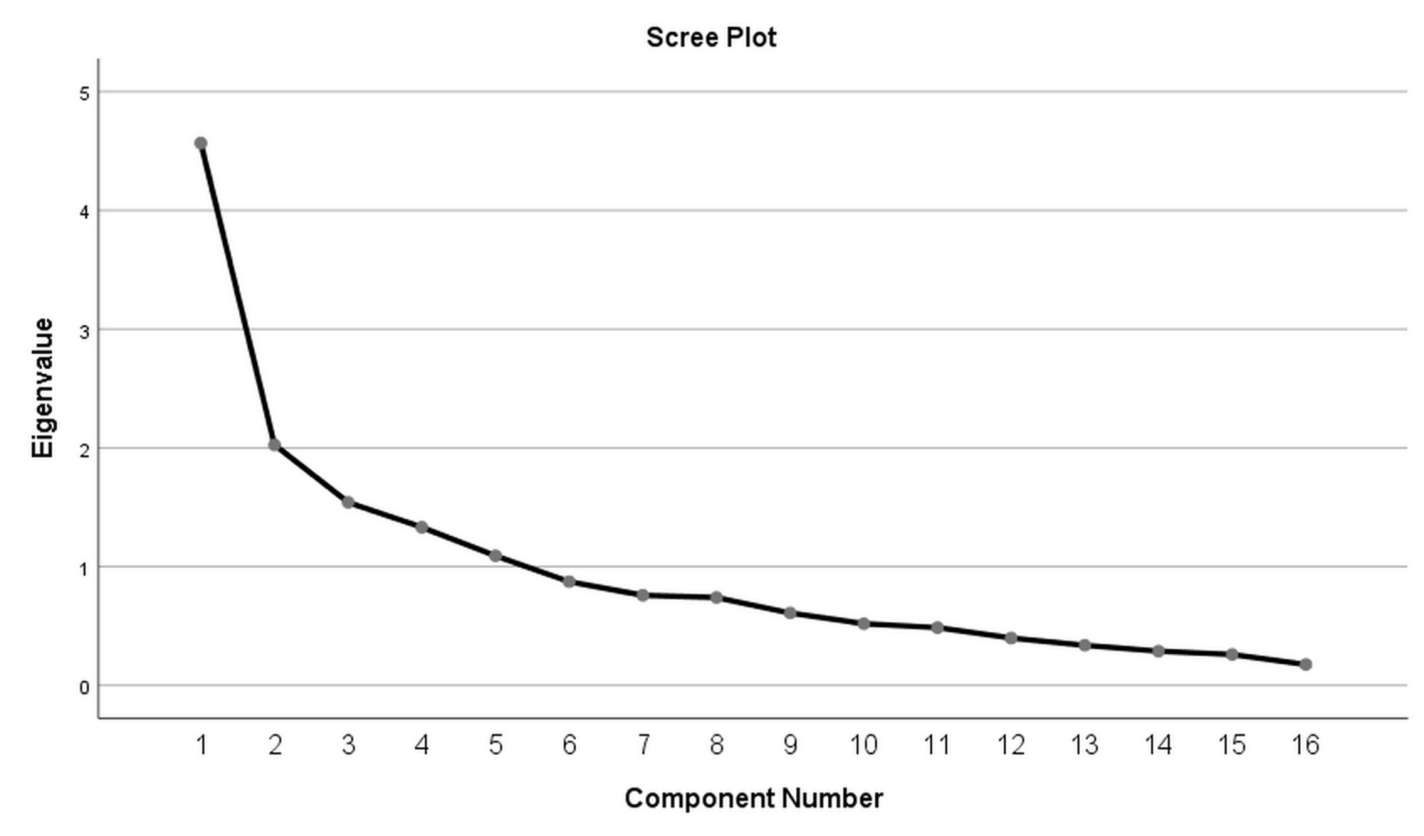
Scree plot

The scree plot for the analysis is depicted in Figure 1. The first four components are clearly over the 1.0 Eigenvalue mark, while a fifth component is very near the limit. However, producing a factor analysis with five components leads to two components with a single item, clearly a sub-optimal solution, so the four components option was confirmed as the best solution.

Item loadings on each component are presented in Table 1. There is very little overlap between the components, with only item 16 having similar loads with components 1 and 4 (0.470 vs 0.469). Resulting components were studied for interpretability of the factors.

**Table 1:** Item loadings on each factor

Rotated Component Matrix				
	Component			
	1	2	3	4
1. If I take my medication every day, I feel confident that my blood pressure is under control			0,815	
2. I think I contribute to the improvement of my blood pressure when I take my medication every day			0,828	
3. It happens that I am not sure whether I have taken my tablets	0,743			
4. During holidays or weekends I sometimes forget to take my medication	0,762			
5. I dislike taking medication every day	0,556			
6. I think it is not healthy for your body to take medication every day	0,470			0,469
7. I take special care to do enough exercise to reduce the risk of getting cardiovascular diseases		0,712		
8. I eat less salt in order to avoid cardiovascular diseases		0,884		
9. I feel better taking medication every day			0,425	-0,358
10. The pros of taking medication weigh up against the cons			0,608	
11. I have a busy life; that is why I sometimes forget to take my medication	0,746			
12. I find it hard to stick to my daily regimen of medication taking	0,705			
13. When my blood pressure is under control during my medical checkups, I want to take less medication				0,877
14. I am afraid of side effects	0,583			
15. I eat less fat in order to avoid cardiovascular diseases		0,806		
16. I gather information about possibilities to solve health problems	0,494			
Extraction Method: Principal Component Analysis. Rotation Method: Varimax with Kaiser Normalization.				

The first component explained the largest percentage (22.142%) of the variance and included items 3, 4, 5, 6, 11 and 12, which focus on the regularity of daily medication intake, and two items (14 and 16) that relate to the knowledge of medication side effects and general knowledge. The second and third components explained similar percentages (14.075% and 13.933%, respectively). The second component included items 7, 8 and 15, which relate to taking care about avoiding cardiovascular disease through other means than medication (daily exercise and dietary practices). The third component included items 1, 2, 9 and 10, which relate to positive feelings from medication adherence. The fourth component explained 8.997% and included item 13, while there was also an almost equally high loading on item 6 as with the first component. Item 13 demonstrates a tendency for non-adherence when blood pressure is under control.

Total cumulative percentage of explained variance from the extracted components was 59.148% and is presented in Table 2.

**Table 2:** Percentage of explained variance after the component extraction

Component	Initial Eigenvalues			Rotation Sums of Squared Loadings		
	Total	% of Variance	Cumulative %	Total	% of Variance	Cumulative %
1	4,566	28,54	28,54	3,543	22,14	22,14
2	2,025	12,65	41,19	2,252	14,07	36,21
3	1,541	9,63	50,82	2,229	13,93	50,15
4	1,331	8,32	59,14	1,440	8,99	59,14

Internal consistency of the identified scales was assessed by the Cronbach’s alpha coefficient.

First component’s Cronbach’s alpha equals 0.824 when item 16 is included and 0.828 when it is not included. Second component’s Cronbach’s alpha equals 0.777, while the third component’s Cronbach’s alpha equals 0.688. If we include item 16 to the fourth component (along with item 9), then Cronbach’s alpha equals 0.486. We should note that Cronbach's alpha is not computed with only a single item and that two items may be too low a number to take this result into account.

Comparing the item structure from this Greek translation to those from the original MUAH-16, we can find both differences and similarities.

The original MUAH-16 similarly had four components (subscales). Subscale 1, named “positive attitude towards health care and medication” includes items 3, 5, 7 and 35 and is identical to the third component in our analysis. Subscale 2 (named “lack of discipline”) includes items 23, 24, 26 and 36 and resembles, to a large extent, the first component of our analysis (four out of six common items). Subscale 3 (named “aversion towards medication”) includes items 9, 13, 14 and 16 and is comprised of the other items of our first component and the items from the fourth component. Finally, Subscale 4 (named “active coping with health problems”) includes items 20, 21, 22 and 39 and shares three out of four items with our second component.

The computation of Cronbach's alpha scores for the original subscales showed that the first subscale’s Cronbach’s alpha equals 0.688, the second subscale’s Cronbach’s alpha equals 0.832, the third subscale's Cronbach’s alpha equals 0.53 and the fourth subscale's Cronbach’s alpha equals 0.635. When comparing those values with the values from the revised structure, we can see that they are generally lower, except for the fourth component.

Correlations between the proposed subscales of the MUAH-16, the total score and age of the subjects are presented in Table 3. The correlations between the subscales are in the expected directions. The only statistical significance in the age correlations is that of a negative correlation for Subscale 3 to age, denoting that those subjects of older age were less likely to have aversion to taking medication.

**Table 3:** Subscale intercorrelations and correlation with age of the subjects with statistical significance values ^a^ Correlation is significant at the 0.05 level (2-tailed), ^b^ Correlation is significant at the 0.01 level (2-tailed).

		Subscale 1	Subscale 2	Subscale 3	Subscale 4	Age
Subscale 1: positive attitude towards health care and medication	Spearman r_s_	-	.082	-.247^a^	.286^b^	-.014
	p		.420	.013	.004	.890
Subscale 2: lack of discipline	Spearman r_s_	.082	-	.221^a^	-.187	-.040
	p	.420		.027	.063	.696
Subscale 3: aversion towards medication	Spearman r_s_	-.247^a^	.221^a^	-	-.374^b^	-.228^a^
	p	.013	.027		.000	.022
Subscale 4: active coping with health problems	Spearman r_s_	.286^b^	-.187	-.374^b^	-	.059
	p	.004	.063	.000		.563
Total score	Spearman r_s_	.356^b^	.453^b^	.688^b^	.088	-.186
	p	.000	.000	.000	.38	.064
Age	Spearman r_s_	-.014	-.040	-.228^a^	.059	-
	p	.890	.696	.022	.563	

We compared the two sexes as to the scores in the proposed subscales with the Mann-Whitney Z statistic. There were no statistically significant differences between the sexes (p > .05 in all comparisons).

## Discussion

We have translated, culturally adapted and validated the MUAH-16 questionnaire for use in the Greek population according to the established WHO guidelines. We tested the Greek version factorial invariance with factor analysis and examined its internal consistency. The four subscales that were formed were highly similar to those of the original MUAH-16, since one subscale was identical (Subscale 1) and there were only small differences regarding the item structure of the other subscales. Hence, we may conclude that the Greek translation of the MUAH-16 is a good match for the original version with small, cultural differences. More research is needed in order to validate the proposed revised internal structure with a larger sample.

A secondary finding in our sample was that age was inversely correlated with aversion towards medication (p = 0,022) and was close to being statistically significantly correlated with the total adherence score (p = 0,064). This means that older subjects have less aversion to taking medications, and probably reflects the fact that older patients are receiving their medication for a longer period of time since as age increases, there is an increased likelihood of receiving medication for any number of chronic ailments (hypertension, diabetes mellitus, hyperlipidemia, etc.). As a result, older patients may have come to recognize the need to do so while younger patients are not used to taking medication. Younger patients are also at an earlier stage of the disease and may be reluctant to acknowledge the fact that they are expected to take medications for the rest of their lives, or they may have in their minds that only older people need constant medication. This finding is in accordance with the literature since a recent systematic review and meta-analysis of patients with long-term conditions showed that higher adherence was associated with stronger perceptions of the necessity of treatment, and fewer concerns about treatment [19]. Thus, older individuals are more in terms with the necessity of receiving regular medication than younger individuals.

A limitation of this study is the small sample of Greek patients that was evaluated during this translation and cultural validation process. A larger number of patients would be required to assess factorial validity with a confirmatory factor analysis in the future.

## Conclusions

The Greek translation of the MUAH-16 is a good match for the original version, with minor cultural differences, and can be used as a valid instrument for identifying causes of non-adherence to antihypertensive treatment in Greek patients, thus opening the way for more targeted, more optimized and, hence, more successful interventions in this specific population. A simple-to-use and easy-to-score self-report questionnaire on medication adherence, such as the MUAH-16, can help the clinician determine whether there is an increased need for patient education on the long-term importance of adherence to antihypertensive medication. Future studies that employ this questionnaire can explore intrinsic factors for non-adherence, such as personality structure and other health-related beliefs.
